# *Lactiplantibacillus koreensis* sp. nov. and *Lactiplantibacillus kimchii* sp. nov., isolated from kimchi, a traditional Korean fermented food

**DOI:** 10.71150/jm.2507007

**Published:** 2025-11-30

**Authors:** Min Ji Lee, Jisu Lee, Sohee Nam, Mi-Ja Jung, Yeon Bee Kim, Yujin Kim, Jeong Ui Yun, Seong Woon Roh, Tae Woong Whon, Che Ok Jeon, Se Hee Lee

**Affiliations:** 1 Kimchi Functionality Research Group, World Institute of Kimchi, Gwangju 61755, Republic of Korea; 2 Department of Life Science, Chung-Ang University, Seoul 06974, Republic of Korea; 3 Department of Integrative Biological Sciences, Chosun University, Gwangju 61452, Republic of Korea; 4 Department of Life Science, Chosun University, Gwangju 61452, Republic of Korea; 5 Microbiome Research Team, LISCure Biosciences Inc., Seongnam 13486, Republic of Korea

**Keywords:** *Lactiplantibacillus koreensis*, *Lactiplantibacillus kimchi*, strain CBA3605^T^, strain CBA3606^T^, kimchi, lactic acid bacteria

## Abstract

Two Gram-stain-positive, facultatively anaerobic, rod-shaped, and non-motile lactic acid bacterial strains, designated as strains CBA3605^T^ and CBA3606^T^, were isolated from kimchi, a traditional Korean fermented food. Both strains were oxidase- and catalase-negative, non-spore-forming, non-hemolytic, and non-gas-producing. Optimal growth conditions for the two strains were observed at 30°C, pH 5.0, and 0% NaCl. The two genomes were composed of a circular chromosome and three plasmids and the DNA G + C content of 43.0%, respectively. Strains CBA3605^T^ and CBA3606^T^ were most closely related to *Lactiplantibacillus* (*Lp*.) *pingfangensis* 382-1^T^ with 16S rRNA sequence similarity of 99.4% and 99.1%, respectively. However, the orthologous average nucleotide identities between CBA3605^T^ and CBA3606^T^ were 91.7%, and those with strain 382-1^T^ were 76.9% and 76.5%, respectively. Digital DNA–DNA hybridization values between CBA3605^T^ and CBA3606^T^ were 45.0%, and those with strain 382-1^T^ were 21.4% and 21.0%, respectively. The major fatty acids detected in both strains included C_16:0_, C_18:1_ ω9c, and summed features 7 (C_19:1_ ω7c, C_19:1_ ω6c, C_19:0_ cyclo ω10c, and/or C_19:0_ ω6c). The peptidoglycan of both strains CBA3605^T^ and CBA3606^T^ contained meso-diaminopimelic acid and was classified as A4α type (L-Lys–D-Asp). In polar lipid analyses, only strain CBA3605^T^ contained aminophosphoglycolipid, which was absent in CBA3606^T^, although both strains harbored same major polar lipids (diphosphatidylglycerol, phosphatidylglycerol, and phosphatidylethanolamine). Based on phenotypic, phylogenetic, genomic, biochemical, and chemotaxonomic analyses, strains CBA3605^T^ and CBA3606^T^ represent two novel species of the genus *Lactiplantibacillus*, for which the names *Lactiplantibacillus koreensis* sp. nov. and *Lactiplantibacillus kimchii* sp. nov. are proposed, with CBA3605^T^ (= KACC 81073BP^T^ = JCM 37965^T^), and CBA3606^T^ (= KACC 81074BP^T^ = JCM 37966^T^) as the type strains.

## Introduction

In 2020, the genus *Lactobacillus* (*Lb*.) was reclassified into 25 distinct genera based on a polyphasic taxonomic approach (Zheng et al., 2020). One of the newly established genera is *Lactiplantibacillus* (*Lp*.), characterized as facultatively anaerobic, homofermentative, Gram-stain-positive, rod-shaped, non-motile, non-spore-forming, and urease-negative. They are able to ferment a wide range of carbohydrates. Most species metabolize phenolic acids through esterase, decarboxylase, and reductase activities, while some exhibit pseudocatalase activity or reduce nitrate under specific conditions. *Lactiplantibacillus* species possess relatively large genomes, with sizes generally between 2.63 and 3.65 Mbp, and their DNA G + C contents range from 42.9 to 48.7 mol%. The name *Lactiplantibacillus* is derived from Latin: *lactis* (milk), *planta* (plant), and *bacillus* (rod), reflecting its origin as a group of rod-shaped lactic acid bacteria (LAB) associated with plants. In line with its name, most species of *Lactiplantibacillus* have been initially isolated from fermented or fermenting plant-based foods, such as Ghanaian fermenting cocoa beans (De Bruyne et al., 2009), Chinese fermented radish pickles (Mao et al., 2015), Chinese fermented cabbage pickles (Liu and Gu, 2019), and Russian sauerkraut (Heng et al., 2023). *Lp. plantarum* DSM 20174^T^, the type species of *Lactiplantibacillus*, was also isolated from pickled cabbages (Orla-Jensen, 1919). However, no novel species of this genus has been reported from kimchi, which is one of the most well-known plant-based fermented foods.

In this study, we report two novel strains, CBA3605^T^ and CBA3606^T^, isolated from kimchi and belonging to the genus *Lactiplantibacillus*. Based on a polyphasic taxonomic approach, including phylogenetic, genomic, physiological, biochemical, and chemotaxonomic analyses, we propose that strains CBA3605^T^ and CBA3606^T^ represent two novel *Lactiplantibacillus* species.

## Materials and Methods

### Isolation and culture conditions

Strains CBA3605^T^ and CBA3606^T^ were isolated from Korean cabbage kimchi in Gwangju, Republic of Korea. Kimchi soup samples were filtered using sterile stomacher filter bags (Nasco) and then serially diluted in 0.85% (w/v) saline solution (3M^TM^). The diluted samples were spread onto de Man, Rogosa, and Sharpe (MRS; BD Difco) agar (Lee et al., 2020) and incubated at 30°C for 2 days. Single colonies were streaked at least three times onto fresh MRS agar to ensure purity. The identified strains, CBA3605^T^ and CBA3606^T^, were preserved at –80°C in 10% (v/v) skim milk for further experiments. The two strains were deposited in the Korean Agricultural Culture Collection (accession numbers KACC 81073BP^T^ and 81074BP^T^) and the Japan Collection of Microorganisms (JCM; accession numbers JCM 37965^T^ and 37966^T^). For taxonomic comparison, phylogenetically related type strains were obtained from the National Collection of Industrial Food and Marine Bacteria (NCIMB) and the Deutsche Sammlung von Mikroorganismen und Zellkulturen (DSMZ): *Lp. daoliensis* NCIMB 15181^T^, *Lp. nangangensis* NCIMB 15186^T^, *Lp. pingfangensis* NCIMB 15187^T^, and *Lp. brownii* DSM 116485^T^. All six strains were cultured under identical conditions for genomic, physiological, biochemical, and chemotaxonomic characterization. To compare their characteristics with *Lp. plantarum* DSM 20174^T^, the type species of *Lactiplantibacillus*, publicly available data from a previous study were used (Curk et al., 1996).

### 16S rRNA gene-based phylogenetic analysis

The 16S rRNA gene sequences of strains CBA3605^T^ and CBA3606^T^ were amplified using PCR pre-mix (Bioneer) and the universal bacterial primers 27F (5'-AGA GTT TGA TCC TGG CTC AG-3′) and 1492R (5′-TAC GGY TAC CTT GTT ACG ACT T-3′) (Lane, 1991). The PCR products were purified and subsequently sequenced to obtain high-quality sequences using a combination of universal bacterial primers, including 27F, 519F, 800R, and 1492R. The 16S rRNA gene sequence similarities between the two strains and validly published type strains were determined using the EzBioCloud server (Yoon et al., 2017). The sequences were aligned using the ClustalW algorithm implemented in SeqMan Pro (DNASTAR) (Burland, 1999) without considering secondary structure, and the phylogenetic trees were constructed using MEGA11 software based on the neighbor-joining (NJ), maximum likelihood (ML), and maximum parsimony (MP) algorithms (Tamura et al., 2021). Bootstrap values were calculated from 1,000 replications. Evolutionary distances were computed using the Kimura two-parameter method (Felsenstein, 1985). *Levilactobacillus tangyuanensis* 137-3^T^ (GenBank accession number MK110859) was used as the outgroup in the phylogenetic trees.

### Genome-based analysis

Whole-genome sequencing of strains CBA3605^T^ and CBA3606^T^ was performed at Macrogen (Republic of Korea) using combined sequencing technologies. Long-read and short-read sequencing were conducted using the PacBio Sequel IIe platform with a 15 kb SMRTbell^TM^ template library and the Illumina HiSeq X Ten platform, respectively. PacBio reads were de novo assembled using the Microbial Assembly application in SMRT Link v11.0, and the assemblies were polished with Pilon v1.21 using the Illumina reads to correct base errors, misassemblies, and fill gaps. Annotation of the complete genome sequences was performed using the NCBI Prokaryotic Genome Automatic Annotation Pipeline (PGAP) (Tatusova et al., 2016). The DNA G + C content of strains CBA3605^T^ and CBA3606^T^ was calculated from their assembled genomes. The completeness and contamination metrics were assessed using CheckM v1.2.3 (Parks et al., 2015). Functional annotation of genes was conducted using the standalone eggNOG-mapper v2 (Cantalapiedra et al., 2021) with default settings, based on the Clusters of Orthologous Groups (COG) database. The proportion of genes assigned to each COG category was calculated as a percentage. To investigate the genomic differences between strains CBA3605^T^ and CBA3606^T^, Kyoto Encyclopedia of Genes and Genomes (KEGG) pathway analysis was performed. The Blast KEGG Orthology And Links Annotation (BlastKOALA) was used for the functional annotation of the predicted proteins in both strains (Kanehisa et al., 2016). Orthologous average nucleotide identity (OrthoANI) and digital DNA–DNA hybridization (dDDH) values were calculated from the whole-genome sequences using the Orthologous ANI Tool (OAT; https://www.ezbiocloud.net/tools/orthoani) (Lee et al., 2016) and the Genome-to-Genome Distance Calculator version 3.0 (GGDC 3.0; https://ggdc.dsmz.de/ggdc.php) (Goris et al., 2007), respectively. To construct a phylogenomic tree, the genome sequences of strains CBA3605^T^, CBA3606^T^, and closely related *Lactiplantibacillus* species with publicly available genomes were retrieved from NCBI GenBank (https://www.ncbi.nlm.nih.gov/genome/). The genome sequence of *Lp. plantarum* DSM 20174^T^ was retrieved and included in the comparative analysis. A phylogenomic tree was reconstructed using the up-to-date bacterial core gene (UBCG2) pipeline (Kim et al., 2021), which employs a set of 100 conserved bacterial core genes. The core genes were extracted from the genomes of interest, individually aligned, and concatenated to construct a maximum-likelihood (ML) phylogenetic tree implemented in FastTree (Price et al., 2009).

### Physiological, biochemical, and chemotaxonomic analyses

Strains CBA3605^T^, CBA3606^T^, and four closely related type strains were pre-cultured on MRS agar at 30°C for 2 days for physiological, biochemical, and chemotaxonomic analyses. All six strains were subsequently subjected to experiments, conducted at 30°C for 2 days unless otherwise specified. Optimal growth conditions for pH, temperature, and NaCl concentration were assessed using MRS medium. The pH tolerance was tested in MRS broth adjusted to pH 3.0–9.0 at 1.0 unit intervals and incubated for 2 days. MRS broths at pH 3.0–6.0, pH 6.0–8.0, and pH 8.0–9.0 were prepared using citric acid/sodium citrate, Na_2_HPO_4_/NaH_2_PO_4_ and Tris–HCl buffers (Gomori, 2010), respectively. The pH levels were readjusted after autoclaving at 121°C for 15 min. Temperature tolerance was tested on MRS agar at 5–50°C in 5°C intervals, and NaCl tolerance was tested at 0–10% NaCl in 2% intervals. Anaerobic growth was examined on MRS agar under anaerobic conditions using a BBL GasPak Plus system (BD Biosciences) for 2 days at 30°C (Agarwal et al., 2006). Motility was assessed in MRS broth containing 0.2% agar for 2 days at 30°C (Cappuccino and Sherman, 2008) and spore formation was evaluated by exposing the strains to 100°C for 30 min using a heating block, followed by incubation on MRS agar at 30°C for 2 days to assess the growth of heat-resistant spores (Kent et al., 2016). Gas production from glucose was tested in MRS broth using Durham tubes. Gram staining was performed using a Gram stain kit (Becton, Dickinson and Company) according to the manufacturer’s instructions. Cell morphology of strains CBA3605^T^ and CBA3606^T^ was observed using a scanning electron microscope (SEM, Verios 5 XHR, Thermo Fisher Scientific). For the sample preparation, cells were washed three times with sterile distilled water, dropped onto silicon wafers, and air-dried for 3 days. The dried samples were coated with a 20 nm platinum layer and observed by SEM. Biochemical features, including carbon source utilization, were analyzed using the API 50 CHL (bioMérieux) system, following the manufacturer’s instructions. Catalase test was conducted using an oxygen bubble formation in a 3% (v/v) hydrogen peroxide solution (Whittenbury, 1964). Oxidase activity was determined based on color changes observed on oxidase test discs. Hemolytic activity was evaluated on MRS agar supplemented with 5% (v/v) sheep blood. Tellurite tolerance was tested using MRS agar supplemented with 0.04% (w/v) potassium tellurite (K_2_TeO_3_, Sigma-Aldrich). The isomeric composition of lactic acid was determined enzymatically using the D/L-lactate test kit (Megazyme). Cellular fatty acids were extracted from strains at the same growth phase and analyzed following the protocol of the Sherlock Microbial Identification System (MIDI) (Sasser, 1990). Fatty acid methyl esters were separated by gas chromatography (model 6890, Hewlett Packard), and identified using the MOORE6 database in the Sherlock software version 6.0B (Miller and Berger, 1985). Peptidoglycan amino acids were extracted and hydrolyzed in 6 N HCl at 121°C for 15 min. The hydrolysates were subjected to thin-layer chromatography (TLC) as described previously (Schleifer and Kandler, 1972), and amino acid components were visualized with ninhydrin. Polar lipids were obtained from cells harvested during the exponential growth phase and analyzed by TLC according to standard procedures (Minnikin et al., 1977). Specific lipid classes were detected using following reagents: 10% ethanolic molybdophosphoric acid for total polar lipids, ninhydrin for aminolipids, the Dittmer-Lester reagent for phospholipids, and α-naphthol/sulfuric acid for glycolipids.

## Results and Discussion

### 16S rRNA gene-based phylogeny

**Fig. 1.**
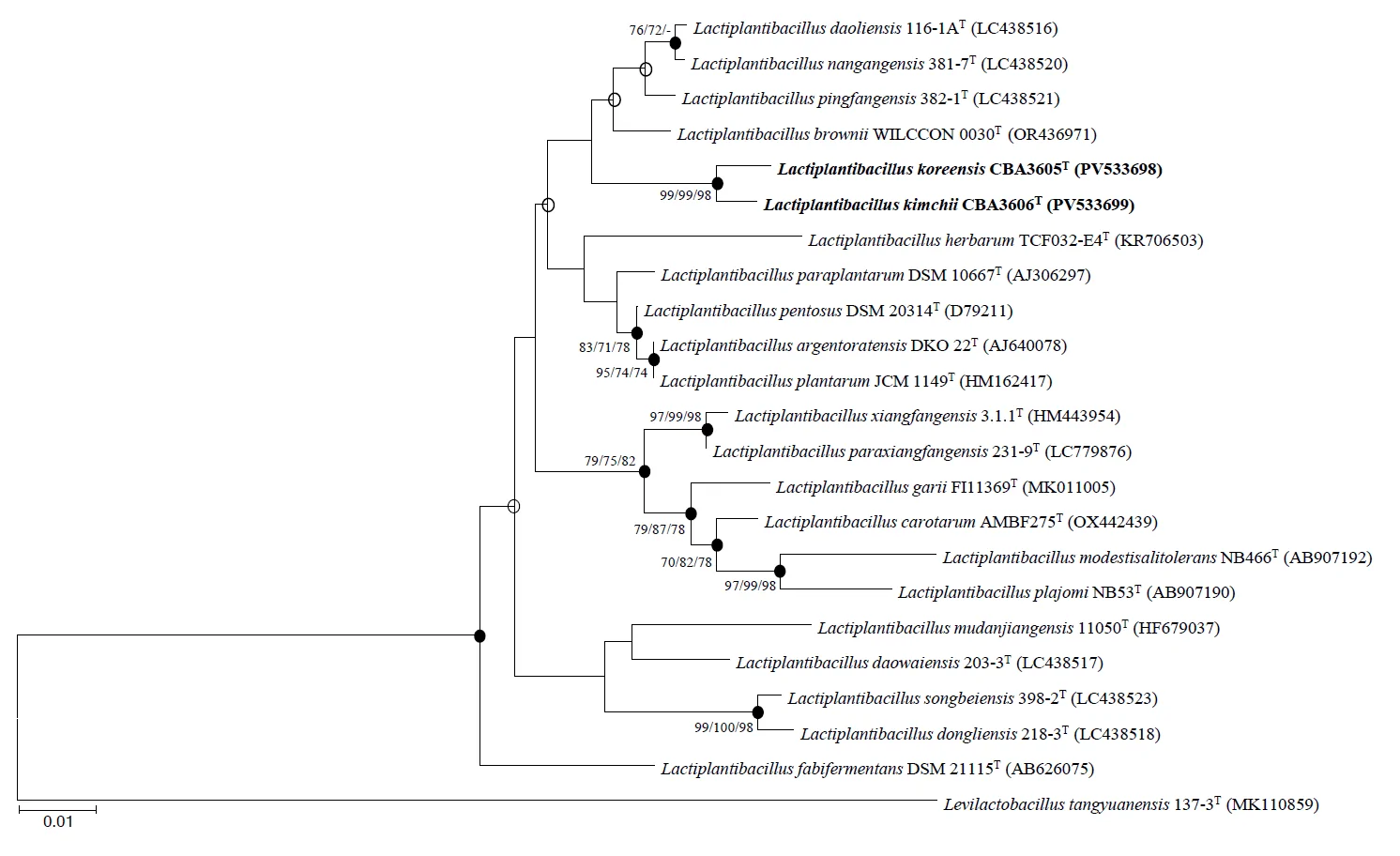
Phylogenetic tree constructed based on 16S rRNA gene sequences of strains CBA3605^T^, CBA3606^T^, and other type strains of the genus *Lactiplantibacillus* using the neighbor-joining method. Bootstrap values based on 1,000 replicates are shown at the nodes, but only those exceeding 70.0% are displayed for the neighbor-joining, maximum-parsimony, and maximum-likelihood methods. Filled circles indicate nodes supported by all three treeing methods. *Levilactobacillus tangyuanensis* 137-3^T^ (MK110859) was used as the outgroup. Scale bar represents 0.01 substitutions per nucleotide position.

Phylogenetic trees based on 16S rRNA gene sequences were constructed using the NJ (Fig. 1), MP (Fig. S1A), and ML (Fig. S1B) algorithms. All trees consistently indicated that strains CBA3605^T^ and CBA3606^T^ belong to the genus *Lactiplantibacillus* and are most closely related to *Lp. daoliensis* 116-1A^T^, *Lp. nangangensis* 381-7^T^, *Lp. pingfangensis* 382-1^T^, and *Lp. brownii* WILCCON 0030^T^. Their 16S rRNA gene sequence similarities with strain CBA3605^T^ were 99.1%, 99.0%, 99.4%, and 99.1%, while those with strain CBA3606^T^ were 98.8%, 98.7%, 99.1%, and 98.9%, respectively. Interestingly, similar to strains CBA3605^T^ and CBA3606^T^, which were isolated from kimchi, a traditional Korean fermented food, the four closely related type strains were also isolated from fermented foods such as pickles and sauerkraut (Heng et al., 2023; Liu and Gu, 2019). The similarity between strains CBA3605^T^ and CBA3606^T^ was 99.8%. Based on these phylogenetic relationships, further analyses, including genomic, physiological, biochemical, and chemotaxonomic characterization, were conducted using all six strains.

### Genomic analysis

**Fig. 2.**
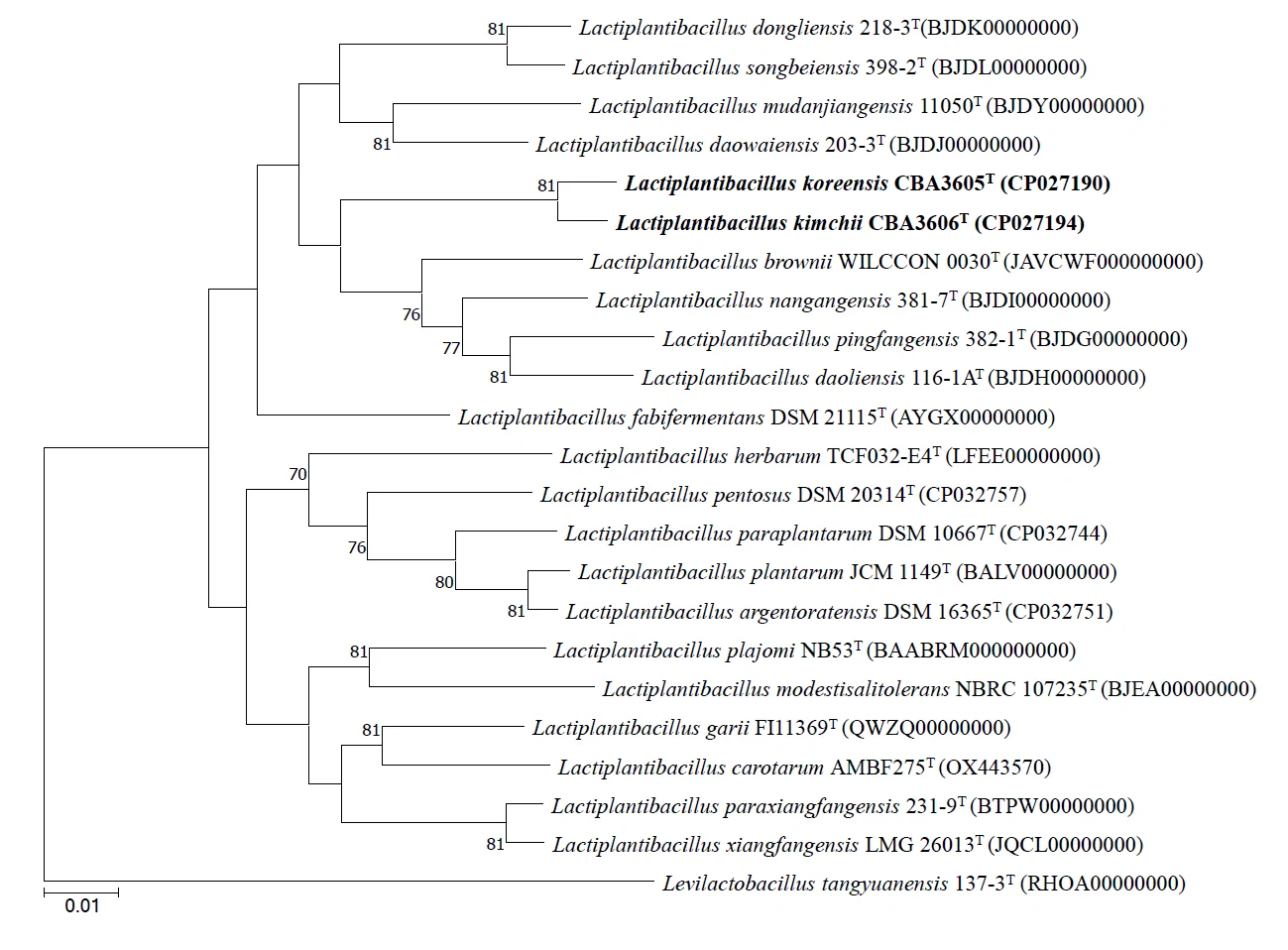
Phylogenetic tree constructed based on whole genome sequences of strains CBA3605^T^, CBA3606^T^, and other type strains of the genus *Lactiplantibacillus* using the maximum-likelihood methods. Bootstrap values based on 1,000 replicates are shown at the nodes, but only those exceeding 70.0% are displayed. *Levilactobacillus tangyuanensis* 137-3^T^ (RHOA00000000) was used as the outgroup. Scale bar represents 0.1 substitutions per sequence position.

Consistent with the 16S rRNA gene-based phylogeny, whole genome phylogenetic analysis revealed that strains CBA3605^T^ and CBA3606^T^ are most closely related to *Lp. daoliensis* 116-1A^T^, *Lp. nangangensis* 381-7^T^, *Lp. pingfangensis* 382-1^T^, and *Lp. brownii* WILCCON 0030^T^, while forming a distinct clade separate from the four *Lactiplantibacillus* type strains (Fig. 2). The general genomic features of these two strains, their closely related type strains, and *Lp. plantarum* DSM 20174^T^ (type species) are summarized in Table 1. Notably, the genome sizes of strains CBA3605^T^ and CBA3606^T^ were approximately 2.4–2.5 Mbp, which are smaller than those of other species that range from 2.6 to 3.3 Mbp. Strains CBA3605^T^ and CBA3606^T^ also harbored lower total gene and protein-coding gene counts compared to *Lp. nangangensis* 381-7^T^, *Lp. pingfangensis* 382-1^T^, *Lp. brownii* WILCCON 0030^T^, and *Lp. plantarum* DSM 20174^T^. However, the numbers of 5S rRNA, 16S rRNA, 23S rRNA, and tRNA genes in strains CBA3605^T^ and CBA3606^T^ were higher than those of *Lp. daoliensis* 116-1A^T^, *Lp. nangangensis* 381-7^T^, and *Lp. pingfangensis* 382-1^T^. Although the two strains exhibited different genomic features compared to their closest species, appropriate thresholds are required to determine their distinction. Moreover, such thresholds are necessary to evaluate whether strains CBA3605^T^ and CBA3606^T^ themselves represent different species. To clarify this point, OrthoANI and dDDH analyses were performed (Table 2). OrthoANI and dDDH values between the two strains were 91.7% and 45.0%, respectively, which are below the commonly accepted species thresholds of 95–96% for OrthoANI (Kim et al., 2014) and 70% for dDDH (Goris et al., 2007), indicating that the strains represent distinct species. Additionally, both strains showed considerably low OrthoANI and dDDH values when compared with the type strains of *Lactiplantibacillus*, further supporting their novelty.

**Fig. 3.**
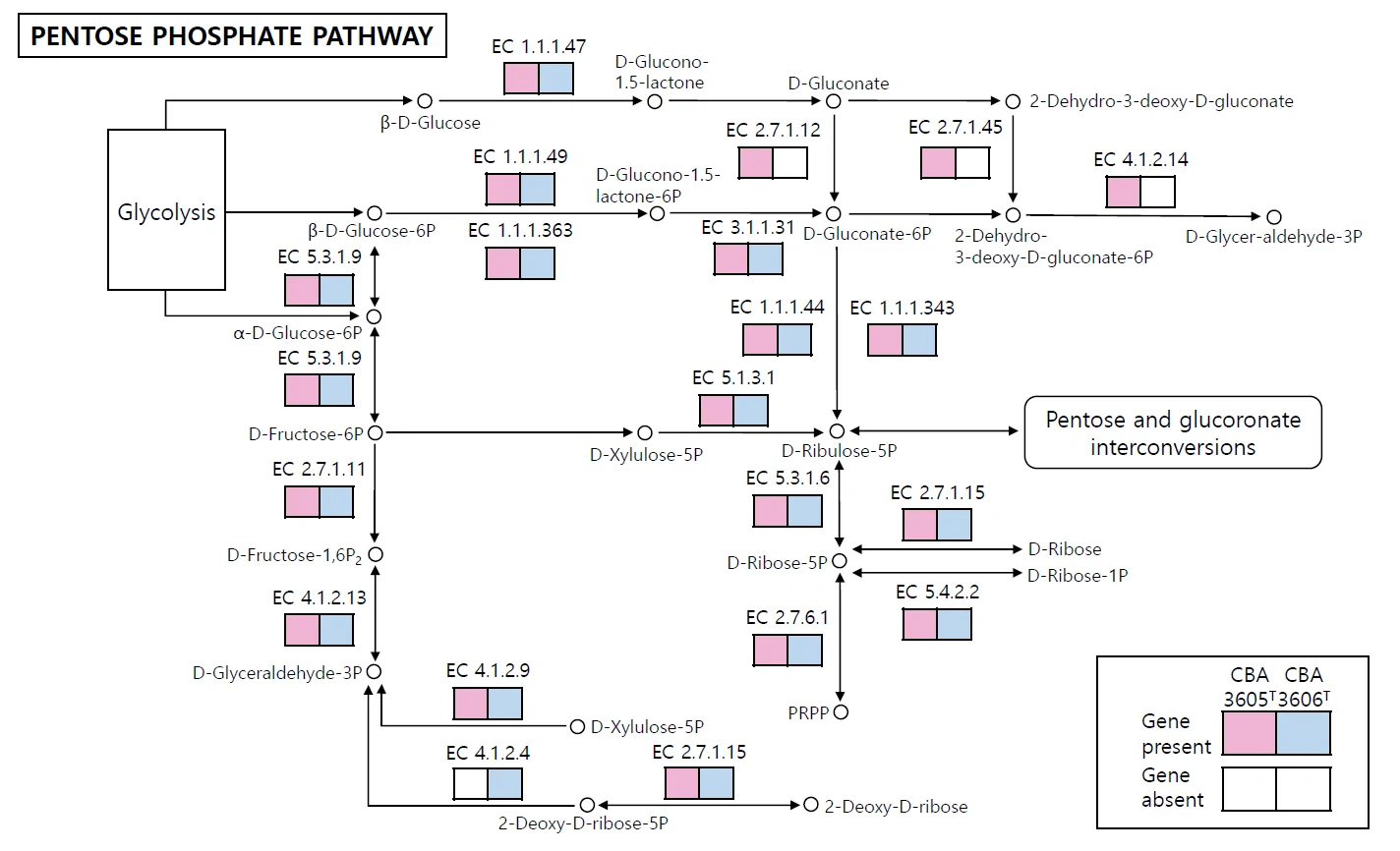
Reconstructed pentose phosphate pathway of strains CBA3605^T^ and CBA3606^T^. Pink and blue boxes indicate genes present in strain CBA3605^T^ and CBA3606^T^, respectively, while white boxes denote the absence of the corresponding genes. Genes absent in both strains are not shown.

The COG category distributions of strains CBA3605^T^, CBA3606^T^, *Lp. daoliensis* 116-1A^T^, *Lp. nangangensis* 381-7^T^, *Lp. pingfangensis* 382-1^T^, *Lp. brownii* WILCCON 0030^T^, and *Lp. plantarum* DSM 20174^T^ are summarized in Table S1. In all seven strains, the largest proportion of genes was assigned to category K (transcription), ranging from 10.1% to 12.3%. In strains CBA3605^T^ and CBA3606^T^, the second most abundant categories were J (translation, ribosomal structure, and biogenesis) and L (DNA replication, recombination, and repair), respectively, while the third most enriched categories were G (carbohydrate transport and metabolism) and J, respectively. This suggests functional divergence between the two strains. We further investigated the distinct genomic features between strains CBA3605^T^ and CBA3606^T^ using KEGG analysis (Fig. 3). In the metabolic pathways, especially in the pentose phosphate pathway, strain CBA3605^T^ harbored the genes encoding gluconokinase (EC 2.7.1.12), 2-dehydro-3-deoxygluconokinase (EC 2.7.1.45), and 2-dehydro-3-deoxyphosphogluconate aldolase (EC 4.1.2.14), which were absent in strain CBA3606^T^. In contrast, the gene encoding deoxyribose-phosphate aldolase (EC 4.1.2.4) was present in strain CBA3606^T^ but absent in strain CBA3605^T^. These findings support the differentiation between strains CBA3605^T^ and CBA3606^T^.

In addition to these genomic differences, KEGG analysis suggested that both strains are homofermentative. They harbored the gene encoding fructose-bisphosphate aldolase (EC 4.1.2.13; locus_tags C5Z25_RS08140 in CBA3605 and C5Z26_RS09410 in CBA3606), a key enzyme present in homofermentative but absent in heterofermentative LAB strains (Chun et al., 2017). To further confirm this, we reconstructed the metabolic pathways from glucose to lactate using strains CBA3605^T^ and CBA3606^T^, together with *Lp. plantarum* DSM 20174^T^ (homofermentative control) and *Leuconostoc* (*Leu*.) *mesenteroides* ATCC 8293^T^ (heterofermentative control) (Fig. S2). The reconstructed pathway clearly showed that strains CBA3605^T^ and CBA3606^T^ harbored the complete set of glycolytic genes, including phosphofructokinase-1 (EC 2.7.1.11) and fructose-biphosphate aldolase (EC 4.1.2.13), consistent with homofermentative metabolism. In contrast, the heterofermentative strain *Leu. mesenteroides* ATCC 8293^T^ lacked these key genes, relying instead on the phosphoketolase pathway (Wang et al., 2021). These results demonstrate that strains CBA3605^T^ and CBA3606^T^ are homofermentative members of the genus *Lactiplantibacillus*.

Lastly, considering that the genomes of strains CBA3605^T^ and CBA3606^T^ were complete sequences, we further analyzed their plasmid features using PGAP (Table S2). Both strains possessed three plasmids, but the total plasmid sizes and gene numbers in strain CBA3605^T^ were smaller than those in strain CBA3606^T^. Although most plasmid-borne genes were shared between the two strains, comparative analysis of plasmid sequences revealed 12 unique genes in CBA3605^T^ and 28 unique genes in CBA3606^T^, respectively (Table S3).

Taken together, the 16S rRNA gene sequence and whole-genome analyses suggest that strains CBA3605^T^ and CBA3606^T^ represent novel species within the genus *Lactiplantibacillus*. Further physiological, biochemical, and chemotaxonomic analyses were carried out to support this taxonomic classification.

### Physiological, biochemical, and chemotaxonomic analyses

Tables 3 and 4 presents the phenotypic characteristics of strains CBA3605^T^ and CBA3606^T^, and their closely related type strains. All six strains shared identical growth conditions at 30°C and 0% (w/v) NaCl, whereas their optimal pH values differed. Strains CBA3605^T^, CBA3606^T^, *Lp. nangangensis* NCIMB 15186^T^, and *Lp. pingfangensis* NCIMB 15187^T^ exhibited optimal growth at pH 5, while *Lp. daoliensis* NCIMB 15181^T^ and *Lp. brownii* DSM 116485^T^ exhibited at pH 6 and 7, respectively. All strains formed beige-colored colonies, except *Lp. brownii* DSM 116485^T^, which formed white colonies. Other physiological characteristics were also consistent among the six strains as they were facultatively anaerobic, non-motile, non-spore-forming, oxidase- and catalase-negative, Gram-positive, non-gas-producing, non-hemolytic, and not resistant to tellurite. However, the two strains and their closely related type strains exhibited different acid production patterns. Strains CBA3605^T^ and CBA3606^T^ produced acid from D-ribose, L-rhamnose, D-sorbitol, and gentiobiose, but not from D-raffinose and D-turanose. In contrast, the remaining closely related type strains did not produce acid from L-rhamnose, D-sorbitol. Strain CBA3605^T^ produced acid from D-lactose, but not from methyl-*α*-D-glucopyranoside, *N*-Acetylglucosamine while strain CBA3606ᵀ showed the opposite pattern. Notably, four closely related type strains produced acid from the latter two but not from D-lactose. The remaining close relatives exhibited the same acid production pattern as strain CBA3606^T^. Relevant data of *Lp. plantarum* DSM 20174^T^ from a previous study (Curk et al., 1996) were also included in Table 3 for direct comparison of the six strains with the type species of *Lactiplantibacillus*. In contrast to the other six strains, *Lp. plantarum* DSM 20174^T^ was able to grow in the presence of 8% NaCl but did not survive at pH 4. SEM images showed that strain CBA3605^T^ was rod-shaped and measured approximately 1.8 ± 0.4 µm in length and 0.5 ± 0.1 µm in width. Strain CBA3606^T^ also displayed a rod shaped, with a length of approximately 2.1 ± 0.8 µm and a width of 0.5 ± 0.2 µm (Fig. S3).

The predominant fatty acids of strains CBA3605^T^ and CBA3606^T^, as well as the four closely related type strains, were C_16:0_, C_18:1_ ω9c, and Summed feature 7, which are typical for members of the genus *Lactiplantibacillus*. However, the relative proportions of these fatty acids varied among the strains. In particular, C_18:1_ ω9c was markedly higher in strains, 382-1^T^ and WILCCON 0030^T^ (39.4–40.2%) compared with strains CBA3605^T^, CBA3606^T^, 116-1A^T^, and *Lp. nangangensis* 381-7^T^ (22.0–32.4%). Conversely, Summed feature 7 was most abundant in strains CBA3605^T^ and CBA3606^T^ (31.0–33.8%), but relatively lower in strains 382-1^T^ and WILCCON 0030^T^ (24.1–26.6%). C_16:0_ levels were relatively consistent (17.7–23.1%), although strains CBA3605^T^ and CBA3606^T^ showed slightly higher values than the others. These variations in fatty acid composition provide additional phenotypic evidence to differentiate the novel strains from their closest relatives.

TLC analysis of whole-cell hydrolysates showed a clear spot corresponding to meso-diaminopimelic acid (meso-DAP), which was confirmed by comparison with a standard (Fig. S4). Additional faint spots were observed at higher Rf values that are consistent with lysine and aspartic acid, which were further supported by genome annotation. Genome annotation supported these findings, revealing genes for meso-DAP biosynthesis, including asd (K00133; locus_tag=C5Z25_RS03555 in strain CBA3605^T^; locus_tag=C5Z26_RS04925 in strain CBA3606^T^), dapA (K01610; locus_tag=C5Z25_RS01900 in strain CBA3605^T^; locus_tag=C5Z26_RS03280 in strain CBA3606^T^), and dapF (K01778; locus_tag=C5Z25_RS02640 in strain CBA3605^T^; locus_tag=C5Z26_RS04020 in strain CBA3606^T^), as well as lysA (K01586; locus_tag=C5Z25_RS00380 in strain CBA3605^T^; locus_tag=C5Z26_RS02035 in strain CBA3606^T^) for conversion to L-Lys, and murM (K14188; locus_tag=C5Z25_RS01255 in strain CBA3605^T^; locus_tag=C5Z26_RS02905 in strain CBA3606^T^) and murN (K14189; locus_tag=C5Z25_RS05430 in strain CBA3605^T^; locus_tag=C5Z26_RS05305 in strain CBA3606^T^) for D-Asp cross-bridge formation. Taken together, the peptidoglycan of the strains is classified as A4α type (L-Lys–D-Asp).

Strains CBA3605^T^ and CBA3606^T^ shared the same major polar lipids, namely diphosphatidylglycerol (DPG), phosphatidylglycerol (PG), and phosphatidylethanolamine (PE), whereas distinct differences were observed in the minor components, with variations in both the number and intensity of phospholipid, aminolipid, and glycolipid spots on TLC plates. In particular, CBA3605^T^ contained aminophosphoglycolipid (APGL), which was absent in CBA3606^T^ (Fig. S5).

### Taxonomic conclusion

The results of phylogenetic analyses based on 16S rRNA gene and genome sequences, as well as physiological and chemotaxonomic assessments, clearly demonstrate that strains CBA3605^T^ and CBA3606^T^ belong to the genus *Lactiplantibacillus*. The two strains have genome-based relatedness measures (ANI and dDDH) of 91.7% and 45.0%, respectively, which are well below the generally accepted species thresholds of 95–96% and 70%. Additionally, several distinct phenotypic characteristics suggest that strains CBA3605^T^ and CBA3606^T^ represent different novel species not recognized within the genus *Lactiplantibacillus*. In conclusion, based on the phylogenetic, physiological, and chemotaxonomic evidence, we propose the names *Lactiplantibacillus koreensis* sp. nov. for strains CBA3605^T^ and *Lactiplantibacillus kimchii* sp. nov. for strains CBA3606^T^.

### Description of *Lactiplantibacillus koreensis* sp. nov

*Lactiplantibacillus koreensis* (ko.re.en′sis. N.L. masc. adj. *koreensis*, pertaining to Korea, from where the type strain was isolated).

Cells are Gram-stain-positive, facultatively anaerobic, non-motile, non-hemolytic, and non-spore-forming. Cells are rod-shaped with approximately 1.8 ± 0.4 µm in length and 0.5 ± 0.1 µm in width. Colonies are beige-colored, convex, and smooth. Cells grow at 15–40°C (optimum at 30°C), pH 4.0–7.0 (optimum, pH 5.0), and 0–6.0% NaCl (optimum, 0%). Gas production from glucose, tellurite tolerance, and oxidase and catalase activities are negative. The strain produces D-lactic acid. In the API 50 CHL test, the strain exhibited positive reactions for D-ribose, D-galactose, D-glucose, D-fructose, D-mannose, L-rhamnose, D-mannitol, D-sorbitol, amygdalin, arbutin, esculin ferric citrate, salicin, D-cellobiose, D-maltose, D-lactose, D-sucrose, D-trehalose, gentiobiose, and potassium 2-ketogluconate. Negative reactions are observed for glycerol, erythritol, D-arabinose, L-arabinose, D-xylose, L-xylose, adonitol, methyl-β-D-xylopyranoside, L-sorbose, dulcitol, inositol, methyl-α-D-mannopyranoside, methyl-*α*-D-glucopyranoside, *N*-acetylglucosamine, D-melibiose, inulin, D-melezitose, D-raffinose, starch, glycogen, xylitol, D-turanose, D-lyxose, D-tagatose, D-fucose, L-fucose, D-arabitol, L-arabitol, potassium gluconate, and potassium 5-ketogluconate. Major cellular fatty acids are identified as C_16:0_, C_18:1_ ω9c, and summed features 7 (C_19:1_ ω7c, C_19:1_ ω6c, C_19:0_ cyclo ω10c, and/or C_19:0_ ω6c). The cell-wall peptidoglycan contains meso-diaminopimelic acid and is classified as A4α type (L-Lys–D-Asp). Major polar lipids consist of DPG, PG, and PE. Minor polar lipids include unidentified glycolipid 1 (GL1), unidentified glycolipid 2 (GL2), unidentified glycolipid 3 (GL3), unidentified glycolipid 4 (GL4), unidentified glycolipid 5 (GL5), unidentified glycolipid 6 (GL6), unidentified glycolipid 7 (GL7), unidentified lipid 1 (L1), unidentified lipid 2 (L2), phospholipid 1 (PL1), phospholipid 2 (PL2), phospholipid 3 (PL3), aminophospholipid 1 (APL1), aminophospholipid 2 (APL2), aminophospholipid 3 (APL3), and aminophosphoglycolipid (APGL).

The type strain, CBA3605^T^ (=KACC 81073BP^T^ = JCM 37965^T^), was isolated from cabbage kimchi in Gwangju, Republic of Korea. The whole-genome sequence of the type strain is 2,494,909 bp in length with a DNA G + C content of 43 mol%. GenBank accession numbers for the 16S rRNA gene and the genome sequence of the type strain are PV533698 and CP027190, respectively.

### Description of *Lactiplantibacillus kimchii* sp. nov.

*Lactiplantibacillus kimchii* (kim′chi.i. M.L. gen. n. *kimchii* from kimchi, a Korean fermented vegetable food).

Cells are Gram-stain-positive, facultatively anaerobic, non-motile, non-hemolytic, and non-spore-forming. Cells are rod-shaped with approximately 2.1 ± 0.8 µm in length and 0.5 ± 0.2 µm in width. Colonies are beige-colored, flat, and dry. Cells grow at 15–40°C (optimum at 30°C), pH 4.0–7.0 (optimum, pH 5.0), and 0–6.0% NaCl (optimum, 0%). Gas production from glucose, tellurite tolerance, and oxidase and catalase activities are negative. The strain produces D-lactic acid. In API 50 CHL test, the strain exhibited positive reactions for D-ribose, D-galactose, D-glucose, D-fructose, D-mannose, L-rhamnose, D-mannitol, D-sorbitol, methyl-*α*-D-glucopyranoside, *N*-acetylglucosamine, amygdalin, arbutin, esculin ferric citrate, salicin, D-cellobiose, D-maltose, D-sucrose, D-trehalose, gentiobiose, and potassium 2-ketogluconate. Negative reactions are observed for glycerol, erythritol, D-arabinose, L-arabinose, D-xylose, L-xylose, adonitol, methyl-β-D-xylopyranoside, L-sorbose, dulcitol, inositol, methyl-α-D-mannopyranoside, D-lactose, D-melibiose, Inulin, D-melezitose, D-raffinose, starch, glycogen, xylitol, D-turanose, D-lyxose, D-tagatose, D-fucose, L-fucose, D-arabitol, L-arabitol, potassium gluconate, and potassium 5-ketogluconate. Major fatty acids are identified as C_16:0_, C_18:1_ ω9c, and summed features 7 (C_19:1_ ω7c, C_19:1_ ω6c, C_19:0_ cyclo ω10c, and/or C_19:0_ ω6c). The cell-wall peptidoglycan contains meso-diaminopimelic acid and is classified as A4α type (L-Lys–D-Asp). Major polar lipids consist of DPG, PG, and PE. Minor polar lipids include unidentified glycolipid 1 (GL1), unidentified glycolipid 2 (GL2), unidentified glycolipid 3 (GL3), unidentified glycolipid 4 (GL4), unidentified glycolipid 5 (GL5), unidentified glycolipid 6 (GL6), unidentified glycolipid 7 (GL7), unidentified lipid 1 (L1), unidentified lipid 2 (L2), phospholipid 1 (PL1), phospholipid 2 (PL2), phospholipid 3 (PL3), aminophospholipid 1 (APL1), aminophospholipid 2 (APL2), and aminophospholipid 3 (APL3).

The type strain, CBA3606^T^ (=KACC 81074BP^T^ = JCM 37966^T^), was isolated from cabbage kimchi in Gwangju, Republic of Korea. The whole-genome sequence of the type strain is 2,449,069 bp in length with a DNA G + C content of 43 mol%. GenBank accession numbers for the 16S rRNA gene and the genome sequence of the type strain are PV533699 and CP027194, respectively.

## Figures and Tables

**Table 1.** General genomic features of strains CBA3605^T^, CBA3606^T^, their closely related type strains, and strain DSM 20174^T^ of the genus *Lactiplantibacillus* (*Lp*.)

Characteristic	1^*^	2^*^	3	4	5	6	7
Status^†^ (Number of contigs)	C (4)	C (4)	D (19)	D (39)	D (59)	D (11)	C (2)
Genome size (bp)	2,494,909	2,449,069	2,632,925	2,901,270	2,895,555	3,347,921	3,250,154
DNA G + C content (%)	43.0	43.5	43.5	44.5	44.0	43.5	44.5
Number of genes	2,497	2,493	2,466	2,779	2,733	3,187	3,086
Number of protein-coding genes	2,362	2,330	2,385	2,711	2,656	3,030	2,962
Number of tRNA genes	62	62	50	43	50	63	71
Number of pseudogenes	54	82	25	18	19	74	33
Number of rRNA gene operons (5S, 16S, 23S)	6, 5, 5	6, 5, 5	1, 1, 1	1, 1, 1	1, 2, 1	6, 5, 5	6,5,5
GenBank accession numbers	CP027190–3	CP027194–7	BJDH	BJDI	BJDG	JAVCWF00000000	NZ_CP039121–2
			00000000	00000000	00000000		
Completeness (%)	98.5	99.0	99.4	99.0	99.4	99.0	99.0
Contamination (%)	1.2	1.4	0.7	0.7	0.7	1.1	1.4

Strains: 1, CBA3605^T^; 2, CBA3606^T^; 3, *Lp. daoliensis* 116-1A^T^; 4, *Lp. nangangensis* 381-7^T^; 5, *Lp. pingfangensis* 382-1^T^; 6, *Lp. brownii* WILCCON 0030^T^; 7, *Lp. plantarum* DSM 20174^T^ (type species of the genus *Lactiplantibacillus*).

* Genome sequenced in this study.

† Genome status: C, complete genome sequence; D, draft genome sequence.

**Table 2.** Pair-wise average nucleotide identity (ANI) and digital DNA-DNA hybridization (dDDH) values of strains CBA3605^T^, CBA3606^T^, their closely related type strains, and strain DSM 20174^T^ of the genus *Lactiplantibacillus* (*Lp*.); numbers below and to the left of the diagonal represent ANI values, while those above and to the right of the diagonal represent dDDH values.

	1	2	3	4	5	6	7
1	-	45.00	21.40	21.00	21.30	21.70	20.70
2	91.69	-	21.00	20.90	21.30	21.50	21.00
3	76.92	76.52	-	25.20	25.90	24.70	20.70
4	76.66	76.64	81.76	-	24.40	24.60	21.10
5	76.62	76.65	82.38	80.81	-	24.10	20.60
6	76.85	76.73	80.78	80.98	80.23	-	21.70
7	74.86	74.91	75.03	74.97	74.71	75.12	-

Strains: 1, CBA3605^T^; 2, CBA3606^T^; 3, *Lp. daoliensis* 116-1A^T^; 4, *Lp. nangangensis* 381-7^T^; 5, *Lp. pingfangensis* 382-1^T^; 6, *Lp. brownii* WILCCON 0030^T^; 7, *Lp. plantarum* DSM 20174^T^ (type species of the genus *Lactiplantibacillus*).

**Table 3.** Phenotypic characteristics of strains CBA3605^T^, CBA3606^T^, their closely related type strains, and strain DSM 20174^T^ of the genus *Lactiplantibacillus* (*Lp*.)

Characteristic	1	2	3	4	5	6	7
Colony color	Beige	Beige	Beige	Beige	Beige	White	N.D.
Optimum growth at:							
pH (optimum)	4.0–7.0 (5.0)	4.0–7.0 (5.0)	4.0–7.0 (6.0)	4.0–7.0 (5.0)	4.0–7.0 (5.0)	4.0–7.0 (7.0)	5.0–7.0 (N.D.)
Temperature (optimum, °C)	15–35 (30)	15–35 (30)	15–35 (30)	15–35 (30)	15–35 (30)	15–35 (30)	15–37 (N.D.)
NaCl (optimum, %)	0–6.0 (0)	0–6.0 (0)	0–4.0 (0)	0–6.0 (0)	0–4.0 (0)	0–6.0 (0)	0–8.0 (N.D.)
API 50 CHL test:							
D-ribose	+	+	-	-	+	+	+
L-rhamnose	+	+	-	-	-	-	-
D-sorbitol	+	+	-	-	-	-	+
Gentiobiose	+	+	+	-	+	-	+
D-raffinose	-	-	-	+	-	-	+
D-turanose	-	-	-	-	+	-	+
D-lactose	+	-	-	-	-	-	+
methyl-α-D-glucopyranoside	-	+	+	+	+	+	N.D.
*N*-acetylglucosamine	-	+	+	+	+	+	+

Strains: 1, CBA3605^T^; 2, CBA3606^T^; 3, *Lp. daoliensis* NCIMB 15181^T^; 4, *Lp. Nangangensis* NCIMB 15186^T^; 5, *Lp. pingfangensis* NCIMB 15187^T^; 6, *Lp. brownii* DSM 116485^T^; 7, *Lp. plantarum* DSM 20174^T^ (data for strain from Curk et al., 1996). Data were obtained from this study unless otherwise indicated. Symbols: +, positive; −, negative; N.D., no data

**Table 4.** Cellular fatty acid composition (percentages) of strains CBA3605^T^, CBA3606^T^, and their closely related type strains of the genus *Lactiplantibacillus* (*Lp*.)

Fatty acid	1	2	3	4	5	6
Saturated						
C_10:0_	-	-	-	tr	-	tr
C_12:0_	tr	tr	tr	tr	tr	tr
C_14:0_	tr	tr	tr	tr	tr	tr
C_16:0_	23.13	22.57	17.67	18.69	21.20	18.96
C_17:0_	tr	tr	tr	tr	tr	tr
C_18:0_	1.84	1.95	2.95	3.72	2.11	2.19
C_19:0_	-	-	tr	-	-	-
C_20:0_	tr	tr	20.86	5.72	tr	tr
Unsaturated						
C_17:1_ ω8c	tr	tr	tr	tr	tr	tr
C_18:1_ ω9c	32.40	30.29	21.97	29.87	39.43	40.20
C_20:1_ ω7c	-	-	tr	tr	tr	tr
C_20:1_ ω9c	-	-	tr	tr	-	-
Branched -chain fatty acid						
C_10:0_ iso	tr	tr	-	-	-	-
C_15:0 _iso	tr	-	tr	-	tr	-
C_19:0_ iso	1.49	1.54	1.80	1.88	1.77	1.55
C_19:1 _iso I	tr	-	tr	tr	tr	tr
Hydroxy fatty acids						
C_17:0_ 2OH	2.89	2.70	2.64	3.22	2.94	3.27
Other						
C_16:0_ N alcohol	-	-	-	0.11	-	-
Summed feature						
3	1.73	1.74	1.04	1.13	1.64	1.42
7	30.98	33.79	25.60	29.46	24.09	26.56
8	3.70	3.71	3.95	4.39	4.16	3.93

Strains: 1, CBA3605^T^; 2, CBA3606^T^; 3, *Lp. daoliensis* NCIMB 15181^T^; 4, *Lp. Nangangensis* NCIMB 15186^T^; 5, *Lp. pingfangensis* NCIMB 15187^T^; 6, *Lp. brownii* DSM 116485^T^. All fatty acid data were obtained during this study unless otherwise indicated. Data are expressed as percentages of total fatty acids. Major components (> 10.0%) are highlighted in bold type. Fatty acids amounting to < 1.0% in all strains are not shown. Symbols: tr, trace amount (< 1.0%); −, not detected. Summed feature 3 contains C_16:1_ ω7c and/or C_16:1_ ω6c, Summed feature 7 contains C_19:1_ ω7c, C_19:1_ ω6c, C_19:0_ cyclo ω10c, and/or C_19:0_ ω6c, Summed feature 8 contains C_18:1_ ω7c and/or C_18:1_ ω6c.

## Supplementary Information

Fig. S1. Phylogenetic trees based on 16S rRNA gene sequences of strains CBA3605ᵀ, CBA3606ᵀ, and other type strains of the genus *Lactiplantibacillus*, constructed using (A) maximum-parsimony (MP) and (B) maximum-likelihood (ML) methods. Bootstrap values calculate from 1,000 replicates and are shown at the nodes; only values exceeding 70.0% are displayed. *Oenococcus oeni* JCM 6125^T^ (GenBank accession no. AB022924) was used as the outgroup. Scale bars indicate 20 and 0.02 substitutions per nucleotide position for MP and ML, respectively.

Fig. S2. Reconstructed metaobolic pathway from glucose to lactate fermentation in homofermentative and heterofermentation strains. Our strains (CBA3605^T^ and CBA3606^T^; red), *Lactiplantibacillus* *plantarum* DSM 20174^T^ (homofermentative reference; blue), and *Leuconostoc mesenteroides* ATCC 8293^T^ (heterofermentative reference; green) were compared. Both strains CBA3605^T^ and CBA3606^T^ possessed the complete set of glycolytic genes, including phosphofructokinase-1 (EC 2.7.1.11) and fructose-bisphosphate aldolase (EC 4.1.2.13), similar to DSM 20174^T^, whereas *Leu. mesenteroides* ATCC 8293^T^ lacked these key genes.

Fig. S3. Scanning electron microscopy (SEM) images showing the cellular morphology of strains (A) CBA3605^T^ and (B) CBA3606^T^ grown on MRS agar at 30°C for 2 days. Scale bar 1 μm.

Fig. S4. Thin-layer chromatograms (TLC) of amino acids from the peptidoglycan of CBA3605^T^, CBA3606^T^, and related type strains of the genus *Lactiplantibacillus*. Amino acids were extracted and hydrolyzed by 6 N HCl at 121°C for 15 min, and analyzed by thin-layer chromatography. Strains: 1, CBA3605^T^; 2, CBA3606^T^; 3, *Lp. daoliensis* NCIMB 15181^T^; 4, *Lp. nangangensis* NCIMB 15186^T^; 5, *Lp. pingfangensis* NCIMB 15187^T^; 6, *Lp. brownii* DSM 116485^T^.

Fig. S5. Two-dimensional thin-layer chromatograms (TLC) of the polar lipids of CBA3605^T^ and CBA3606^T^. Solvent systems: (I) chloroform-methanol-water (65:25:4, v/v/v); (II) chloroform-acetic acid-methanol-water (80:12:15:4, v/v/v/v). The TLC plates were sprayed with 10% ethanolic molybdatophosphoric acid, ninhydrin, and Dittmer-Lester reagents for the detection of total polar lipids. Abbreviations: DPG, diphosphatidylglycerol; PG, phosphatidylglycerol; PE, phosphatidylethanolamine; GL, unidentified glycolipid; L, unidentified lipid; PL, phospholipid; APL, aminophospholipid; APGL, aminophosphoglycolipid.

Table S1. Distribution of Clusters of Orthologous Groups (COG) functional categories in strains CBA3605^T^, CBA3606^T^, their closely related type strains, and strain DSM 20174^T^ of the genus *Lactiplantibacillus* (abbreviated as *Lp*.). The highest, second-highest, and third-highest gene counts for each strain are indicated in bold. Strains: 1, CBA3605^T^; 2, CBA3606^T^; 3, *Lp. daoliensis* 116-1A^T^; 4, *Lp. nangangensis* 381-7^T^; 5, *Lp. pingfangensis* 382-1^T^; 6, *Lp. brownii* WILCCON 0030^T^; 7, *Lp. plantarum* DMS 20174^T^ (type species of the genus *Lactiplantibacillus*)

Table S2. Summary of plasmids in strains CBA3605^T^ and CBA3606^T^

Table S3. Identified unique genes in respective plasmids in strains CBA3605^T^ and CBA3606^T^
