# Supplementary material for: Lactiplantibacillus koreensis sp. nov. and Lactiplantibacillus kimchii sp. nov., isolated from kimchi, a traditional Korean fermented food
Source: J Microbiol. 2025 Nov 30;63(11):e2507007. doi: 10.71150/jm.2507007 (PMC13577789; doi:10.71150/jm.2507007)
Supplement: Supplementary file 1 [file jm-2507007-Supplementary-Fig-S1.pdf]

Fig. S1A

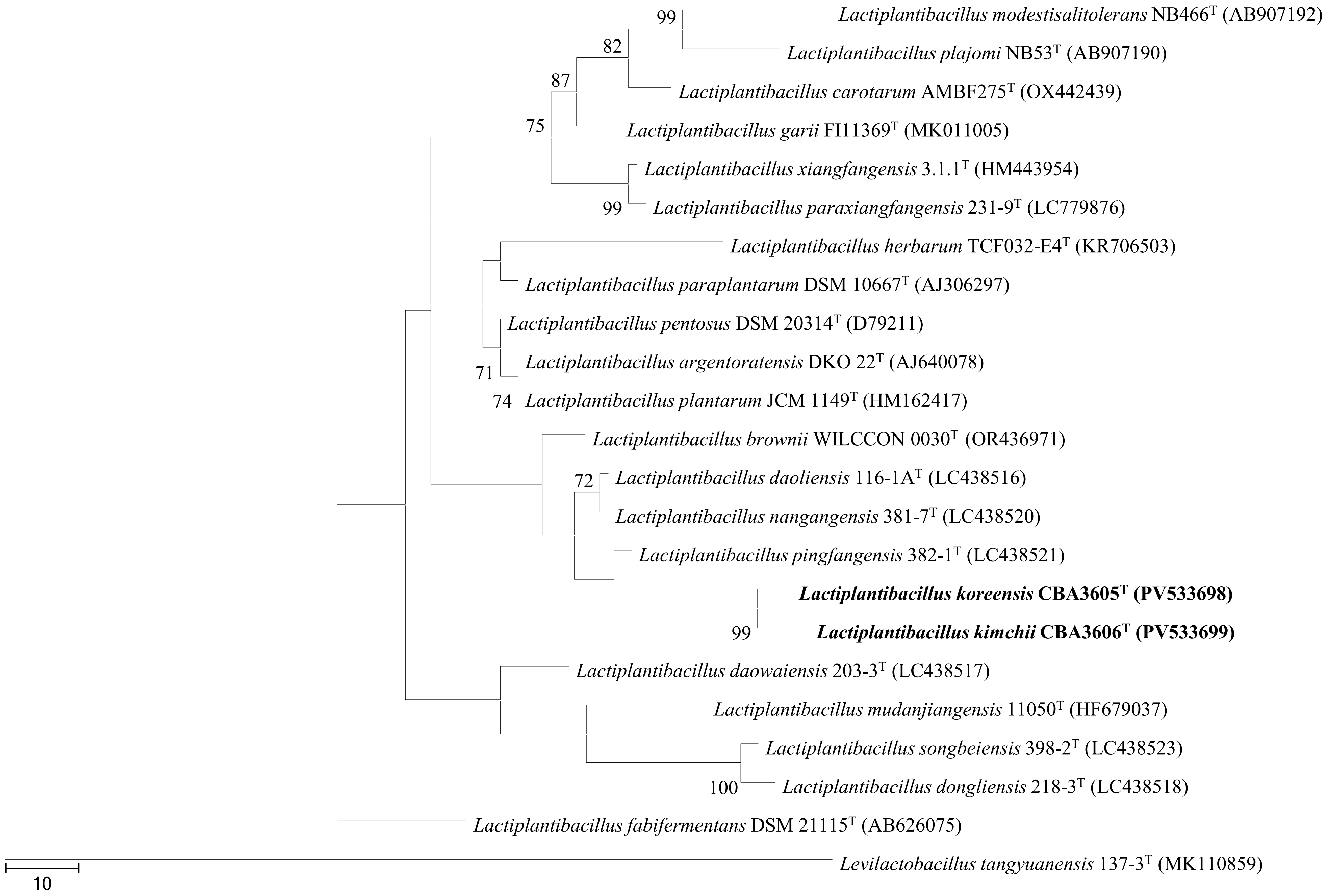

99
Lactiplantibacillus modestisalitolerans NB466T (AB907192)
82
Lactiplantibacillus plajomi NB53T (AB907190)
87
Lactiplantibacillus carotarum AMBF275T (OX442439)
75
Lactiplantibacillus garii FI11369T (MK011005)
Lactiplantibacillus xiangfangensis 3.1.1T (HM443954)
99
Lactiplantibacillus paraxiangfangensis 231-9T (LC779876)
Lactiplantibacillus herbarum TCF032-E4T (KR706503)
Lactiplantibacillus paraplantarum DSM 10667T (AJ306297)
Lactiplantibacillus pentosus DSM 20314T (D79211)
71
Lactiplantibacillus argentoratensis DKO 22T (AJ640078)
74
Lactiplantibacillus plantarum JCM 1149T (HM162417)
Lactiplantibacillus brownii WILCCON 0030T (OR436971)
72
Lactiplantibacillus daoliensis 116-1AT (LC438516)
Lactiplantibacillus nangangensis 381-7T (LC438520)
Lactiplantibacillus pingfangensis 382-1T (LC438521)
Lactiplantibacillus koreensis CBA3605T (PV533698)
99
Lactiplantibacillus kimchii CBA3606T (PV533699)
Lactiplantibacillus daowaiensis 203-3T (LC438517)
Lactiplantibacillus mudanjiangensis 11050T (HF679037)
Lactiplantibacillus songbeiensis 398-2T (LC438523)
100
Lactiplantibacillus dongliensis 218-3T (LC438518)
Lactiplantibacillus fabifermentans DSM 21115T (AB626075)
Levilactobacillus tangyuanensis 137-3T (MK110859)
10

Fig. S1B

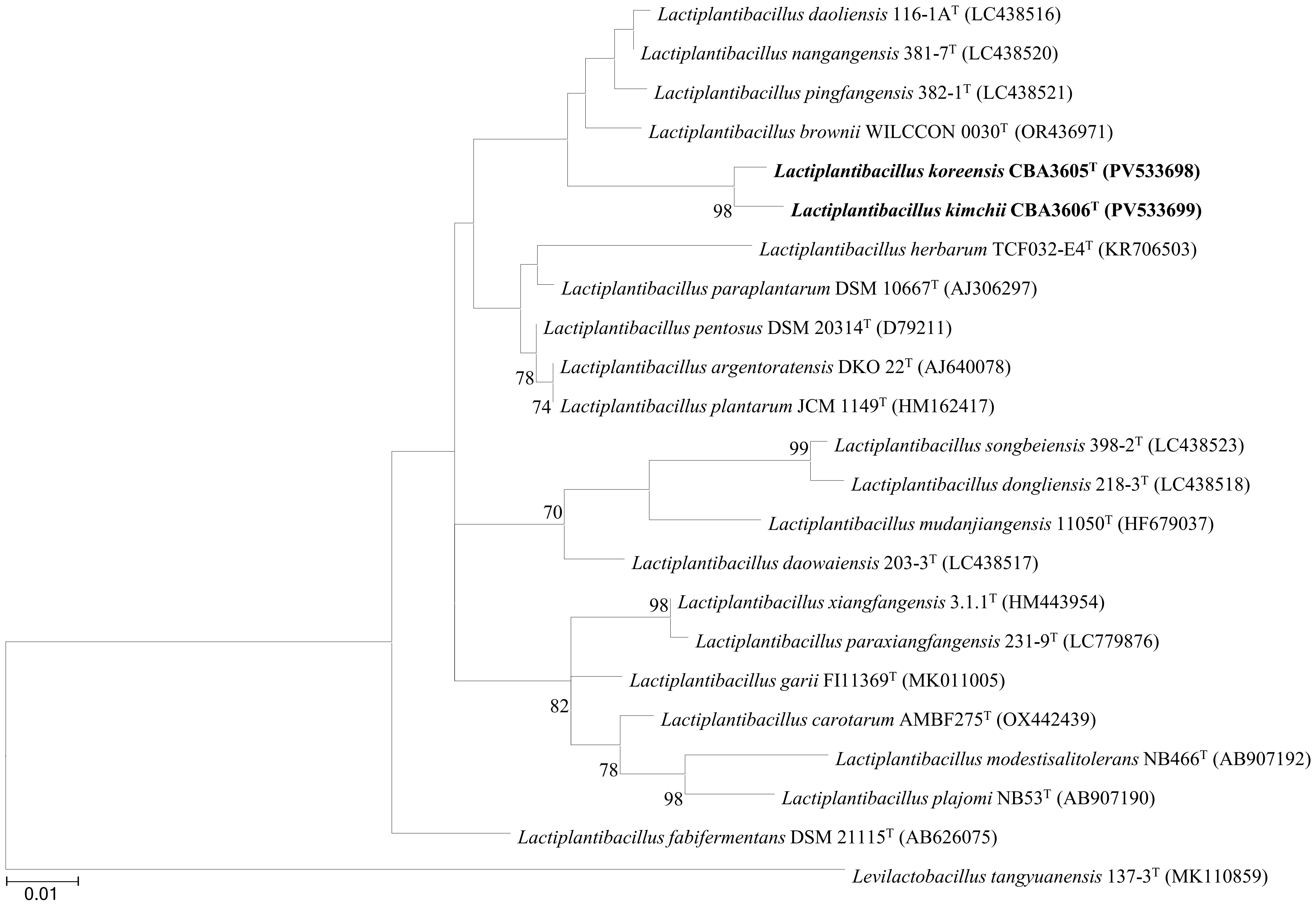
Lactiplantibacillus daoliensis 116-1A^T (LC438516)
Lactiplantibacillus nangangensis 381-7^T (LC438520)
Lactiplantibacillus pingfangensis 382-1^T (LC438521)
Lactiplantibacillus brownii WILCCON 0030^T (OR436971)
Lactiplantibacillus koreensis CBA3605^T (PV533698)
Lactiplantibacillus kimchii CBA3606^T (PV533699)
98
Lactiplantibacillus herbarum TCF032-E4^T (KR706503)
Lactiplantibacillus paraplantarum DSM 10667^T (AJ306297)
Lactiplantibacillus pentosus DSM 20314^T (D79211)
78
Lactiplantibacillus argentoratensis DKO 22^T (AJ640078)
74
Lactiplantibacillus plantarum JCM 1149^T (HM162417)
99
Lactiplantibacillus songbeiensis 398-2^T (LC438523)
Lactiplantibacillus dongliensis 218-3^T (LC438518)
70
Lactiplantibacillus mudanjiangensis 11050^T (HF679037)
Lactiplantibacillus daowaiensis 203-3^T (LC438517)
98
Lactiplantibacillus xiangfangensis 3.1.1^T (HM443954)
Lactiplantibacillus paraxiangfangensis 231-9^T (LC779876)
Lactiplantibacillus garii FI11369^T (MK011005)
82
Lactiplantibacillus carotarum AMBF275^T (OX442439)
78
Lactiplantibacillus modestisalitolerans NB466^T (AB907192)
98
Lactiplantibacillus plajomi NB53^T (AB907190)
Lactiplantibacillus fabifermentans DSM 21115^T (AB626075)
Levilactobacillus tangyuanensis 137-3^T (MK110859)
0.01
