# Supplementary material for: Lactiplantibacillus koreensis sp. nov. and Lactiplantibacillus kimchii sp. nov., isolated from kimchi, a traditional Korean fermented food
Source: J Microbiol. 2025 Nov 30;63(11):e2507007. doi: 10.71150/jm.2507007 (PMC13577789; doi:10.71150/jm.2507007)
Supplement: Supplementary file 2 [file jm-2507007-Supplementary-Fig-S2.pdf]

Fig. S2

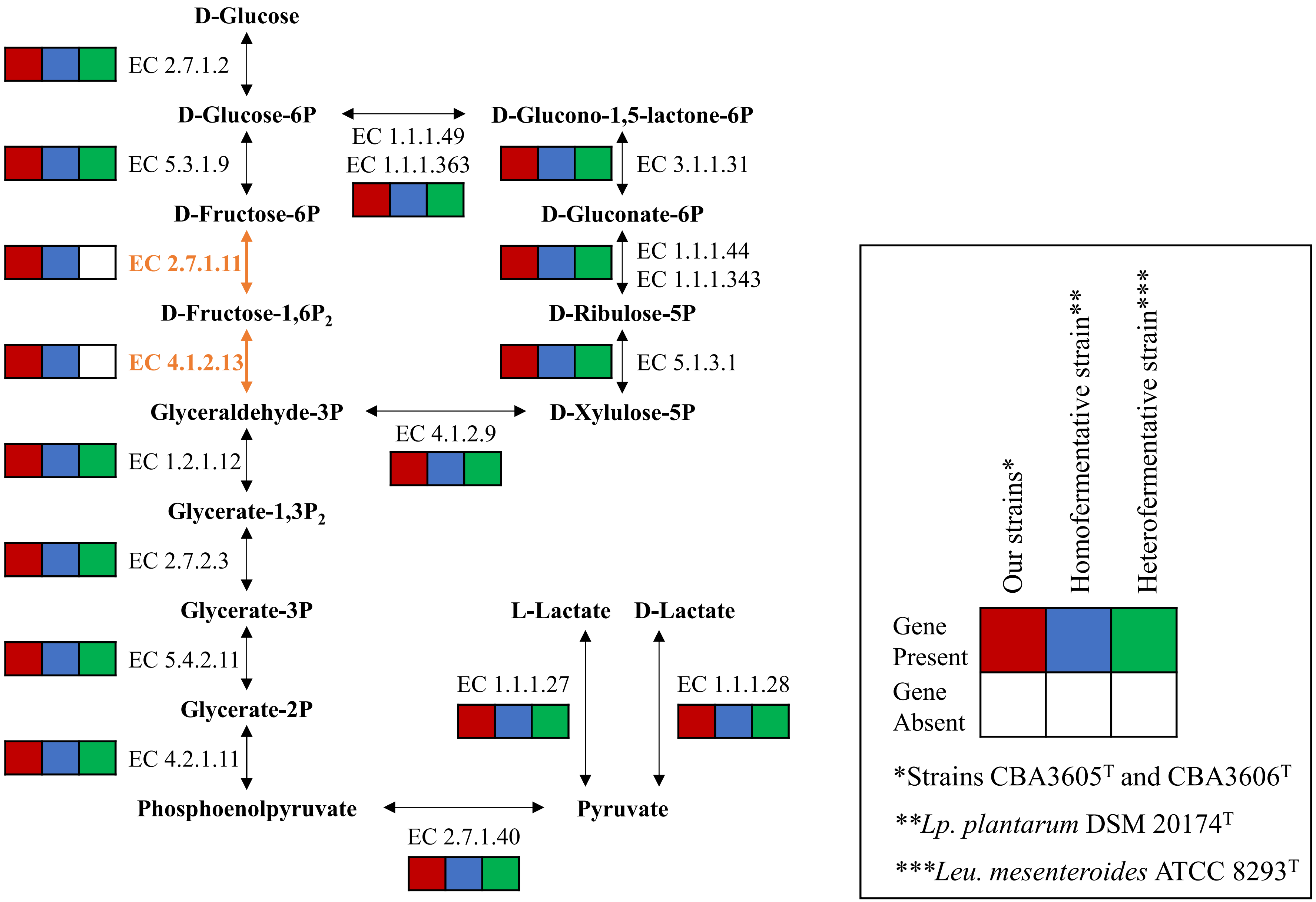

D-Glucose
EC 2.7.1.2
D-Glucose-6P
EC 1.1.1.49
EC 1.1.1.363
D-Glucono-1,5-lactone-6P
EC 5.3.1.9
EC 3.1.1.31
D-Fructose-6P
D-Gluconate-6P
EC 2.7.1.11
EC 1.1.1.44
EC 1.1.1.343
D-Fructose-1,6P$_2$
D-Ribulose-5P
EC 4.1.2.13
EC 5.1.3.1
Glyceraldehyde-3P
D-Xylulose-5P
EC 4.1.2.9
EC 1.2.1.12
Glycerate-1,3P$_2$
EC 2.7.2.3
Glycerate-3P
L-Lactate
D-Lactate
EC 5.4.2.11
EC 1.1.1.27
EC 1.1.1.28
Glycerate-2P
EC 4.2.1.11
Phosphoenolpyruvate
Pyruvate
EC 2.7.1.40
Our strains*
Homofermentative strain**
Heterofermentative strain***
Gene Present
Gene Absent
*Strains CBA3605^T and CBA3606^T
***Lp. plantarum* DSM 20174^T
****Leu. mesenteroides* ATCC 8293^T
