# Supplementary material for: Lactiplantibacillus koreensis sp. nov. and Lactiplantibacillus kimchii sp. nov., isolated from kimchi, a traditional Korean fermented food
Source: J Microbiol. 2025 Nov 30;63(11):e2507007. doi: 10.71150/jm.2507007 (PMC13577789; doi:10.71150/jm.2507007)

Fig. S3

Strain CBA3605ᵀ Strain CBA3606ᵀ

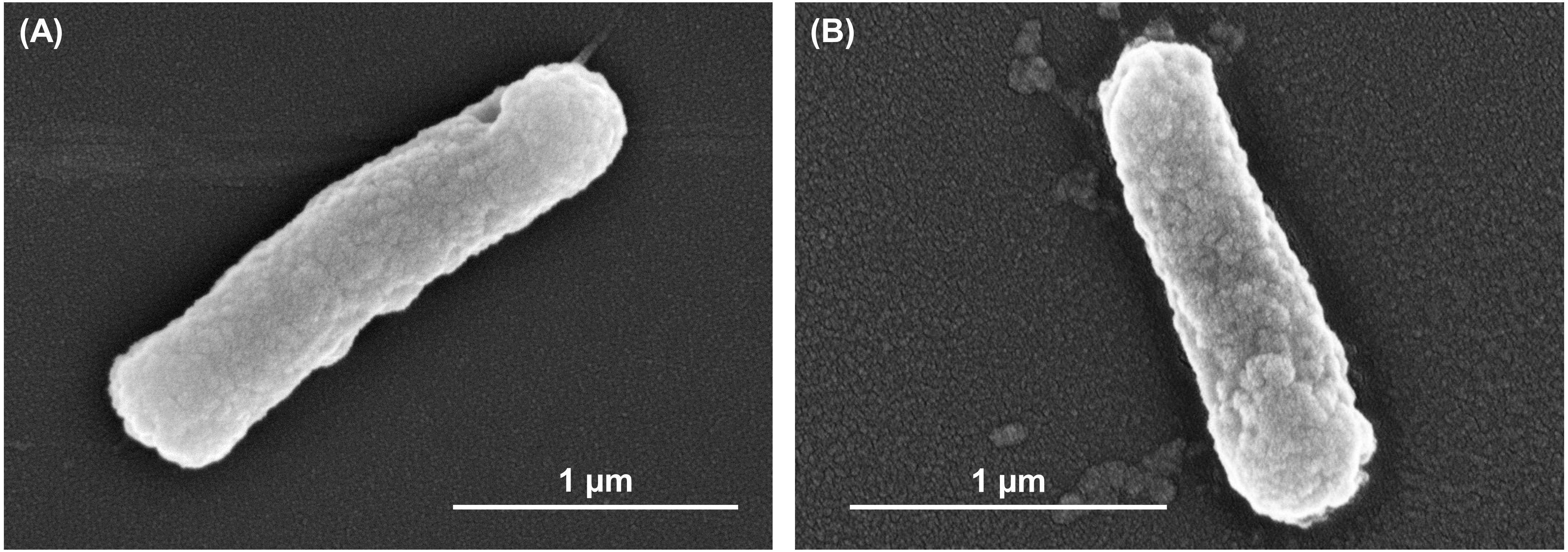

Supplement: Supplementary file 3 [file jm-2507007-Supplementary-Fig-S3.pdf]
