# Supplementary material for: Lactiplantibacillus koreensis sp. nov. and Lactiplantibacillus kimchii sp. nov., isolated from kimchi, a traditional Korean fermented food
Source: J Microbiol. 2025 Nov 30;63(11):e2507007. doi: 10.71150/jm.2507007 (PMC13577789; doi:10.71150/jm.2507007)
Supplement: Supplementary file 5 [file jm-2507007-Supplementary-Fig-S5.pdf]

Fig. S5

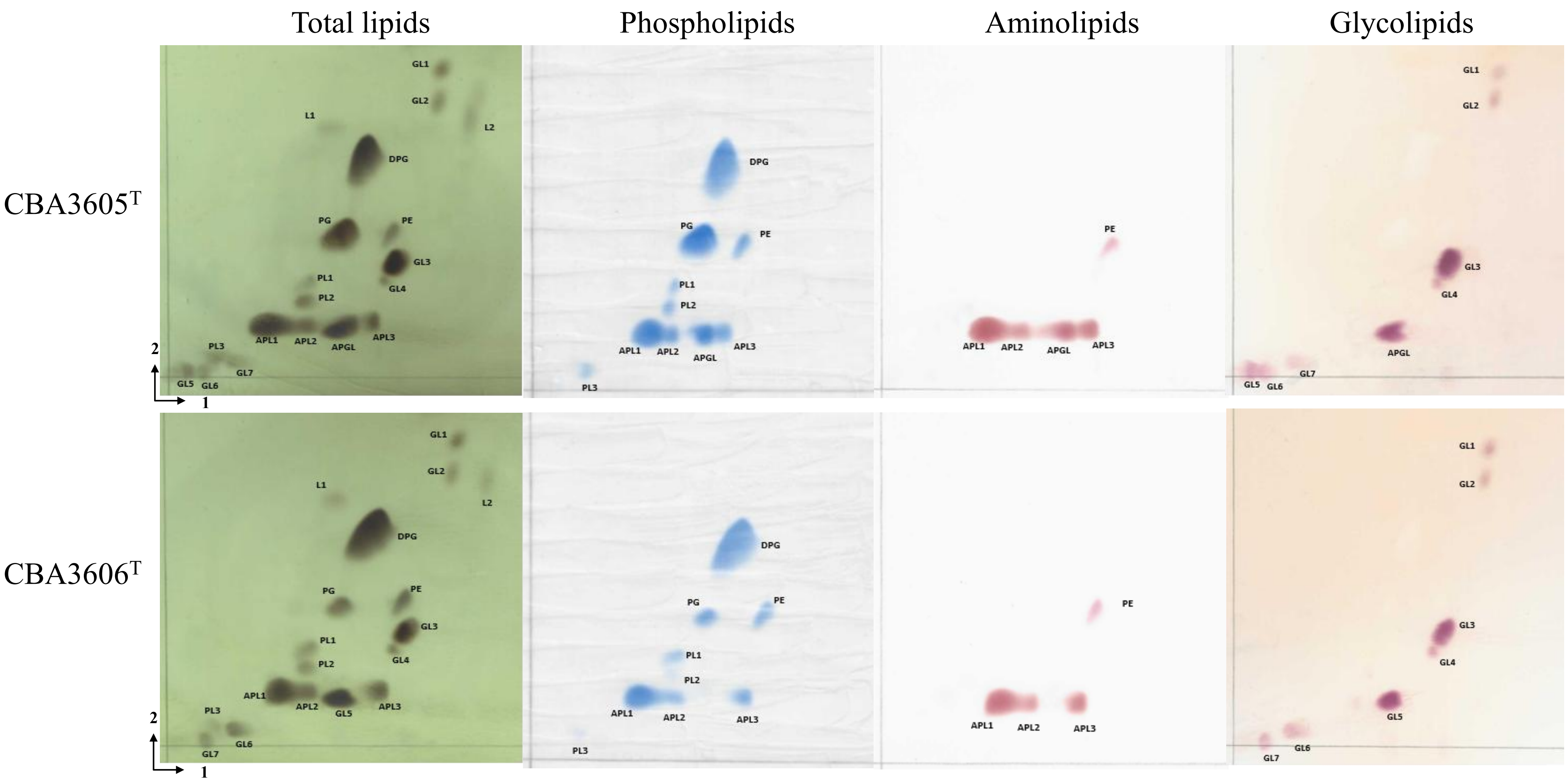

Total lipids
Phospholipids
Aminolipids
Glycolipids
CBA3605T
CBA3606T
GL1
GL2
L1
L2
DPG
PG
PE
GL3
GL4
PL1
PL2
APL1
APL2
APGL
APL3
PL3
GL5
GL6
GL7
2
1
