# Supplementary material for: Lactiplantibacillus koreensis sp. nov. and Lactiplantibacillus kimchii sp. nov., isolated from kimchi, a traditional Korean fermented food
Source: J Microbiol. 2025 Nov 30;63(11):e2507007. doi: 10.71150/jm.2507007 (PMC13577789; doi:10.71150/jm.2507007)
Supplement: Supplementary file 6 [file jm-2507007-Supplementary-Table-S1.pdf]

Table S1. Distribution of Clusters of Orthologous Groups (COG) functional categories in strains CBA3605^T, CBA3606^T, their closely related type strains, and strain DSM 20174^T of the genus *Lactiplantibacillus* (abbreviated as *Lp.*). The highest, second-highest, and third-highest gene counts for each strain are indicated in bold. Strains: 1, CBA3605^T; 2, CBA3606^T; 3, *Lp. daoliensis* 116-1A^T; 4, *Lp. nangangensis* 381-7^T; 5, *Lp. pingfangensis* 382-1^T; 6, *Lp. brownii* WILCCON 0030^T; 7, *Lp. plantarum* DMS 20174^T (type species of the genus *Lactiplantibacillus*)

*COG	1	2	3	4	5	6	7
	No. of genes (%)						
C	87 (4.3)	90 (4.4)	71 (3.5)	81 (3.6)	83 (3.7)	93 (4.0)	116 (4.6)
D	36 (1.8)	38 (1.9)	37 (1.8)	37 (1.7)	37 (1.7)	40 (1.7)	37 (1.5)
E	156 (7.6)	152 (7.4)	166 (8.1)	**186 (8.3)**	**185 (8.3)**	**197 (8.4)**	**215 (8.5)**
F	112 (5.5)	120 (5.9)	117 (5.7)	120 (5.4)	117 (5.3)	127 (5.4)	127 (5.0)
G	**159 (7.8)**	159 (7.8)	**189 (9.2)**	**195 (8.7)**	**193 (8.7)**	**182 (7.8)**	**219 (8.6)**
H	63 (3.1)	61 (3.0)	54 (2.6)	61 (2.7)	62 (2.8)	65 (2.8)	78 (3.1)
I	57 (2.8)	46 (2.2)	43 (2.1)	43 (1.9)	48 (2.2)	49 (2.1)	61 (2.4)
J	**168 (8.2)**	**165 (8.1)**	**167 (8.2)**	167 (7.5)	167 (7.5)	167 (7.2)	174 (6.9)
K	**217 (10.6)**	**207 (10.1)**	**230 (11.2)**	**251 (11.2)**	**273 (12.3)**	**261 (11.2)**	**286 (11.3)**
L	137 (6.7)	**170 (8.3)**	124 (6.1)	154 (6.9)	127 (5.7)	164 (7.0)	141 (5.6)
M	117 (5.7)	117 (5.7)	114 (5.6)	116 (5.2)	128 (5.8)	125 (5.4)	145 (5.7)
N	11 (0.5)	10 (0.5)	10 (0.5)	14 (0.6)	10 (0.4)	12 (0.5)	16 (0.6)
O	44 (2.2)	42 (2.0)	47 (2.3)	44 (2.0)	43 (1.9)	45 (1.9)	48 (1.9)
P	94 (4.6)	96 (4.7)	92 (4.5)	93 (4.2)	98 (4.4)	100 (4.3)	120 (4.7)
Q	12 (0.6)	11 (0.5)	10 (0.5)	10 (0.4)	12 (0.5)	14 (0.6)	17 (0.7)
T	43 (2.1)	42 (2.0)	34 (1.7)	58 (2.6)	38 (1.7)	48 (2.1)	53 (2.1)
U	49 (2.4)	49 (2.4)	51 (2.5)	54 (2.4)	50 (2.3)	59 (2.5)	61 (2.4)
V	39 (1.9)	39 (1.9)	51 (2.5)	59 (2.6)	52 (2.3)	54 (2.3)	59 (2.3)

*The alphabetic codes represent the following COG functional categories: C, energy production and conversion; D, cell division and chromosome partitioning; E, amino acid transport and metabolism; F, nucleotide transport and metabolism; G, carbohydrate transport and metabolism; H, coenzyme metabolism; I, lipid metabolism; J, translation, ribosomal structure, and biogenesis; K, transcription; L, DNA replication, recombination, and repair; M, cell envelope biogenesis, outer membrane; N, cell motility and secretion; O, post-translational modification, protein turnover, and chaperones; P, inorganic ion transport and metabolism; Q, secondary metabolite biosynthesis, transport, and catabolism; T, signal transduction mechanisms; U, intracellular trafficking, secretion, and vesicular transport; V, defense mechanism
