# Supplementary material for: Lactiplantibacillus koreensis sp. nov. and Lactiplantibacillus kimchii sp. nov., isolated from kimchi, a traditional Korean fermented food
Source: J Microbiol. 2025 Nov 30;63(11):e2507007. doi: 10.71150/jm.2507007 (PMC13577789; doi:10.71150/jm.2507007)
Supplement: Supplementary file 7 [file jm-2507007-Supplementary-Table-S2.pdf]

Table S2. Summary of plasmids in strains CBA3605^T and CBA3606^T

**Characteristic**		**CBA3605^T**	**CBA3606^T**
Number of plasmids		3	3
First plasmid	Size (bp)	35,855	51,446
	G + C content (%)	38.5	39.5
	Number of genes	44	56
	GenBank accession numbers	CP027191	CP027195
Second plasmid	Size (bp)	11,850	42,162
	G + C content (%)	44.5	40.0
	Number of genes	13	50
	GenBank accession numbers	CP027192	CP027196
Third plasmid	Size (bp)	9,970	17,059
	G + C content (%)	35.5	40.5
	Number of genes	12	16
	GenBank accession numbers	CP027193	CP027197
