# Supplementary material for: Lactiplantibacillus koreensis sp. nov. and Lactiplantibacillus kimchii sp. nov., isolated from kimchi, a traditional Korean fermented food
Source: J Microbiol. 2025 Nov 30;63(11):e2507007. doi: 10.71150/jm.2507007 (PMC13577789; doi:10.71150/jm.2507007)
Supplement: Supplementary file 8 [file jm-2507007-Supplementary-Table-S3.pdf]

**Table S3.** Identified unique genes in respective plasmids in strains CBA3605$^T$ and CBA3606$^T$

| Strain | No. | Protein name |
|---|---|---|
| CBA3605$^T$ | 1 | DUF2992 domain-containing protein |
| | 2 | HTH domain-containing protein |
| | 3 | IS3-like element IS1163 family transposase |
| | 4 | LtrC |
| | 5 | prevent-host-death family protein |
| | 6 | RelB |
| | 7 | RepB protein |
| | 8 | replication initiation protein |
| | 9 | restriction endonuclease subunit R |
| | 10 | type II toxin-antitoxin system RelE/ParE family |
| | 11 | type II toxin-antitoxin system YafQ family |
| | 12 | XRE family transcriptional regulator |
| CBA3606$^T$ | 1 | alpha/beta hydrolase |
| | 2 | class 1b ribonucleoside-diphosphate reductase |
| | 3 | conjugal transfer protein |
| | 4 | DNA invertase Pin |
| | 5 | DNA topoisomerase III |
| | 6 | DUF536 domain-containing protein |
| | 7 | integrase |
| | 8 | IS110 family transposase |
| | 9 | IS256 family transposase |
| | 10 | IS3 family transposase |
| | 11 | IS5/IS1182 family transposase |
| | 12 | IS982-like element ISLpl4 family transposase |
| | 13 | LysM domain-containing protein |
| | 14 | LysR family transcriptional regulator |
| | 15 | NADH dehydrogenase FAD-containing subunit |
| | 16 | peptidoglycan hydrolase |
| | 17 | phosphohydrolase |
| | 18 | putative sulfate exporter family transporter |
| | 19 | recombinase family protein |
| | 20 | replication protein |
| | 21 | reverse transcriptase |
| | 22 | ribonucleoside-diphosphate reductase |
| | 23 | ribonucleotide reductase assembly protein NrdI |
| | 24 | site-specific DNA-methyltransferase |
| | 25 | theronine dehydrogenase |
| | 26 | thioredoxin |
| | 27 | transposase |
| | 28 | YitT family protein |
